# Does child and adolescent mental health in-service training result in equivalent knowledge gain among cadres of non-specialist health workers in Uganda? A pre-test post-test study

**DOI:** 10.1186/s13033-017-0158-y

**Published:** 2017-08-24

**Authors:** Angela Akol, Joyce Nalugya, Sylvia Nshemereirwe, Juliet N. Babirye, Ingunn Marie Stadskleiv Engebretsen

**Affiliations:** 1 The Global Mental Health Research Group, Center for International Health, University of Bergen, Postboks 7804, N-5009 Bergen, Norway; 2 Makerere University School of Public Health, Kampala, Uganda; 3 Department of Psychiatry, Makerere University College of Health Sciences, Kampala, Uganda; 4 Butabika National Mental Health Referral Hospital, Kampala, Uganda

**Keywords:** Child, Adolescent, Mental health, Training, Non-specialist health worker, Cadre, Primary care

## Abstract

**Background:**

Early identification and management of child and adolescent mental health (CAMH) disorders helps to avert mental illness in adulthood but a CAMH treatment gap exists in Uganda. CAMH integration into primary health care (PHC) through in-service training of non-specialist health workers (NSHW) using the World Health Organisation (WHO) Mental Health Gap Action Programme (mhGAP) Intervention Guide (IG) is a strategy to address this gap. However, results of such training are not supported by information on training development or delivery; and are undifferentiated by NSHW cadre. We aim to describe an in-service CAMH training for NSHW in Uganda and assess cadre-differentiated learning outcomes.

**Methods:**

Thirty-six clinical officers, nurses and midwives from 18 randomly selected PHC clinics in eastern Uganda were trained for 5 days on CAMH screening and referral using a curriculum based on the mhGAP-IG version 1.0 and PowerPoint slides from the International Association of Child and Adolescent Psychiatry and Allied Professions (IACAPAP). The residential training was evaluated through pre- and post- training tests of CAMH knowledge and attitudes using the participants’ post-test scores; and the difference between pre-test and post-test scores. Two-tailed t-tests assessed differences in mean pre-test and post-test scores between the cadres; hierarchical linear regression tested the association between cadre and post test scores; and logistic regression evaluated the relationship between cadre and knowledge gain at three pre-determined cut off points.

**Results:**

Thirty-three participants completed both pre-and post-tests. Improved mean scores from pre- to post-test were observed for both clinical officers (20% change) and nurse/midwives (18% change). Clinical officers had significantly higher mean test scores than nurses and midwives (p < 0.05) but cadre was not significantly associated with improvement in CAMH knowledge at the 10% (AOR 0.08; 95 CI [0.01, 1.19]; p = 0.066), 15% (AOR 0.16; 95% CI [0.01, 2.21]; p = 0.170), or 25% (AOR 0.13; 95% CI [0.01, 1.74]; p = 0.122) levels.

**Conclusion:**

We aimed to examine CAMH learning outcomes by NSHW cadre. NSHW cadre does not influence knowledge gain from in-service CAMH training. Thus, an option for integrating CAMH into PHC in Uganda using the mhGAP-IG and IACAPAP PowerPoint slides is to proceed without cadre differentiation.

**Electronic supplementary material:**

The online version of this article (doi:10.1186/s13033-017-0158-y) contains supplementary material, which is available to authorized users.

## Background

Globally, up to 20% of children and adolescents live with some kind of mental and neurological disorder [1–3]. The burden of CAMH disorders in Uganda has not been accurately estimated but various studies indicate a high prevalence of depression (21%) among school going adolescents [4] and disadvantaged children in four districts (8.6%) [5]. Okello et al. (2007) estimated that approximately 44% of war-affected adolescents in another district suffered from one or more CAMH disorder [6, 7] and a high prevalence of anxiety disorders (26.6%) and adolescent suicidality [8, 9] are documented.

The absence of accurate estimates notwithstanding, a mental health treatment gap is recognized for child and adolescent mental health (CAMH) conditions in low and middle income countries [10–12]. To close the treatment gap, global advocates recommend among other approaches, the integration of CAMH into primary health care (PHC) [3, 13–16], as a task-shifting strategy aimed at improving the human resource availability for the treatment and care of individuals living with mental illness. However, this strategy is challenged by the limited skills and knowledge of non-specialist health workers (NSHW) to provide quality mental health services, a challenge particularly seen with adolescents and children [17–20].

To help correct the skills gap among NSHW, the World Health Organisation (WHO) launched the Mental Health Gap Action Program (mhGAP) in 2008, with the objective of scaling up evidence-based services for the prevention and management of mental, neurological and substance use (MNS) disorders. The attendant mhGAP Intervention Guide (mhGAP-IG) was developed 2 years later as a tool to aid the integration of priority MNS disorders into services provided by NSHW in PHC settings in low- and middle-income countries (LMIC) [21]. As a technical tool, the mhGAP-IG provides simple procedures to aid clinical decision making around a set of priority MNS conditions, including behavioral disorders in children and adolescents. Recent work in Uganda suggests that it is feasible to integrate mental health services into PHC by training NSHW to identify and treat common mental health problems. For example, a 2007 initiative found that mental health training of community health workers and PHC providers from northern Uganda was followed by increased mental health awareness, identification and referral of mental health patients [22, 23]. However, task-shifting interventions such as these to date have all had an adult psychiatry orientation in Uganda.

In-service training of NSHW using the mhGAP-IG falls within the realm of continuing medical education (CME), a globally endorsed strategy for improving human resources for health [3, 16, 21]. In Uganda, CME is policy-endorsed strategy for improving access to CAMH services that is not being implemented at scale [11]. CME may take several forms, ranging from short sessions, computer modules, workshops or courses, each associated with varied learning outcomes [24]. Recent work on CME effectiveness identifies that in the hierarchy of CME teaching and learning, interactive and clinically integrated activities are most effective in impacting learner knowledge, attitudes, behavior and learning outcomes [25, 26]. Such CME initiatives integrate passive classroom teaching with interactive group activities and clinically oriented practicums.

**Fig. 1:**
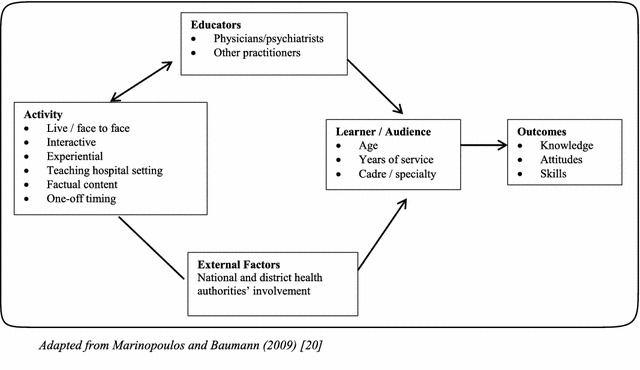
Conceptual model of CME (Adapted from Marinopoulos and Baumann [27])

Conclusions on CME method effectiveness are limited by poor reporting on contextual factors such as the training settings, teacher-learner interactions and educational background of the learners, which may affect delivery of the intervention and the outcomes of training [26]. A conceptual model of CME effectiveness has been proposed by Marinopoulos and Baumann [27] to guide analysis of the covariates of successful CME (Fig. 1). This model advances the idea that a suitable mix of techniques delivered by appropriate educators in a facilitative external environment to motivated learners with the correct social and professional attributes will deliver knowledge, skill and attitudinal outcomes that persist over the long term. Learner characteristics (including cadre and years of service), the nature of the CME activity, the CME educators and external factors all interact to influence learning outcomes. It is not known which of these factors is most important for influencing CAMH learning outcomes for NSHW in Uganda.

Non-specialist health workers are not homogenous and comprise nurses, midwives, medical assistants or clinical officers and community health workers. In Uganda, nurses and midwives receive less exposure than clinical officers to MNS disorders in pre-service training [28, 29], which may affect the outcome of mental health CME. Specifically, it is not clear whether mhGAP training would have the same effect across the different cadres of Ugandan NSHW.

The benefits of mhGAP CME for NSHW are established in the literature [22, 23], but findings are not differentiated by cadre of health worker; and limited information is available on how training is developed, delivered and evaluated [30–33]. Furthermore, the available evidence points to beneficial effects of CME on adult-oriented psychiatry, with scant literature on CAMH training for NSHW. We aim to describe an interactive and clinically-integrated short in-service CAMH training for NSHW in Uganda and to examine how learning outcomes differ by cadre of NSHW. This paper is part of a larger trial which seeks to evaluate the effect of NSHW training on identification of child- and adolescent mental, neurological or substance use disorders through a randomised controlled trial in Uganda.

## Methods

### Study area and study clinics

Eighteen health centers from Mbale and Sironko districts in Eastern Uganda were selected for CAMH training as part of a wider study on access to CAMH services through PHC. These districts were selected for study because they were mhGAP naïve and were in close proximity to the psychiatric unit at Mbale regional referral hospital. Eligible clinics comprised level 3 health centers (HC III) because this is the lowest level at which comprehensive PHC services are provided in Uganda. In addition, the presence of at least four clinical staff at the HC III, made it possible for two staff members to attend CAMH training without disabling service delivery.

A list of all eligible HC III in Mbale and Sironko districts was obtained from the district health offices and 18 clinics were randomly selected using computer generated numbers, by a collaborator who was external to the research team. In randomizing, the clinic list was sorted alphabetically before a random sequence number was generated using the command randomize on stata v.12 (StataCorp. 2011).

### Participants

Participants were clinical officers, nurses and midwives selected from the 18 primary care clinics. The officers in charge of health services in the two districts nominated trainees from each of the selected health facilities, choosing the two most senior NSHW available, with the assumption that senior staff would be able to cascade the training and knowledge to other PHC staff. All selected health facilities sent two NSHWs for the training.

### Intervention

#### Curriculum development

The curriculum was developed at a 2-day workshop attended by mental health professionals from the two national referral hospitals, who had extensive experience with CAMH training of students, mental health professionals and other health workers. During the workshop, the trainers reviewed available training curricula which had been utilized in Uganda and determined, based on their experience, that WHO’s mhGAP-IG [21] modules on developmental disorders and behavioral disorders had appropriate content for training NSHW on CAMH screening, referral and management. Using the mhGAP-IG, a 5-day curriculum (Additional file 1: attachment 1) was developed. The trainers augmented the training with PowerPoint presentations from the International Association of Child and Adolescent Psychiatry and Allied Professions (IACAPAP) textbook [34] to deliver additional detail on conditions included in the mhGAP-IG and on conditions not included in the mhGAP-IG, but which were considered important in the NSHWs’ contexts. The IACAPAP Textbook of Child and Adolescent Mental Health ISBN: 978-0-646-57440-0 is an online textbook first published by the IACAPAP, Geneva in 2012. Designed for mental health professionals and trainees, the textbook delves into the range of possible clinical presentations and treatment options for CAMH disorders.

Once the curriculum had been agreed upon, the trainers reviewed each session and agreed on session flow, session-leads, methods of instruction and interactive activities for each session. For example, it was agreed that to keep the learners engaged, lecture methodology was reserved for the morning sessions and in the afternoons, group work and clinical practice were incorporated. Sessions appropriate for role-play methodology were carefully chosen and additional trainers with session-specific skills sets were identified.

Additionally, the trainers contextualized the content to Uganda. In particular, they prefaced the training with a presentation on Uganda’s mental health status; and illustrative examples in the mhGAP-IG and the IACAPAP slides were reviewed for local applicability and replaced with context-appropriate examples where necessary. Similarly, group discussion topics were contextualised to the Ugandan setting. Due to cost constraints, and basing on the trainers’ experience using these materials with NSHW in other parts of the country, the training curriculum was not pre-tested. However, all training materials were reviewed and approved by the Ministry of Health mental health department prior to training.

#### Training

Thirty-six NSHW were trained. The aim of the training was to equip the NSHW with knowledge and skills in identification, assessment and treatment or referral of children and adolescents with mental health problems, to contribute to early referral of children into care. The 5-day residential training took place in September–October 2015 at the post-graduate center at the main psychiatric referral hospital located in a suburb of the capital city, and was delivered by two psychiatrists and a psychologist with special interest and training in child and adolescent psychiatry and mental health. Together, this team led both classroom and clinical practicum sessions. Classroom training was delivered using a mix of teaching methods including lecture, group discussions, case vignettes and role plays while practicums involved 2 h of clinical demonstration on the children’s ward. The participants all received reference material. Training was evaluated through written pre- and post- tests of knowledge and attitudes towards CAMH.

### Data collection and analysis

#### Instruments

The participant registration form collected participant names, place of work and gender while a participant biodata form collected age, place of work, cadre and years of service. A CAMH knowledge test derived from a standardised assessment designed by the WHO for trainings on the mhGAP IG (Additional file 2: Attachment 2) was administered on day 1 before training and on day 5 after the training to explore NSHW attitudes and knowledge using binary true/false responses and multiple choice questions along the themes in Box 1. The face and construct validity of the assessment tool were determined based on the expert opinion of local professionals and by evidence of its use in the sub-Saharan context [35].

### Box 1: Thematic areas assessed by the pre- and post-training evaluation instrument

- Parental behavior and mental health status
- Childhood behavior
- Depression
- Psychosis
- Epilepsy
- Developmental delay
- Suicidal and self harming behaviour
- Alcohol and substance abuse
- Treatment options for CAMH disorders

#### Measures

For purposes of analysis, nurses and midwives were condensed into one cadre and compared with clinical officers. Participant knowledge scores before and after training were assessed to establish a difference in knowledge gain between the two cadres of NSHW. CAMH knowledge gain was evaluated using two measures. First, the CAMH knowledge possessed by the participants after training was considered (absolute measure), assessed using the post-test score as the outcome variable. Secondly, we considered the change in CAMH knowledge possessed by the participants as a result of training (relative measure). We considered this measure important because it takes participant baseline CAMH knowledge into account and is recommended for understanding longitudinal learning outcomes [36]. To assess relative knowledge gain, the percentage difference in participant pre- and post-tests was the dependent variable. We assessed knowledge gain at three cut-off points (10, 15 and 25%) to detect what cut off point is helpful for assessing knowledge change in the two cadres. A literature search for appropriate cut-off points yielded no information and these cut-off points were determined based on our estimation of three possible, realistic points of knowledge gain for this group of NSHW.

#### Statistical tests

Demographic and occupational information (cadre, number of years in service and place of work) were analyzed descriptively for all participants using proportions and measures of central tendency. Differences in mean pre-test and post-test scores were assessed using the two-tailed student’s *t* test. Outcome measures were assessed in two ways. In the first measure (absolute measure), hierarchical linear regression was applied to test the association between participant cadre and post-test scores, confirming using skewness/kurtosis and Breusch–Pagan/Cook-Weisberg tests that the outcome variable was normally distributed and homoscedastic. In the second measure (relative measure), three cut-off points (10, 15 and 25%) of knowledge gain were set; and logistic regression tested the relationship between NSHW cadre and these cut off points of increase in knowledge gain.

## Results

Analysis was based on the 33 participants who completed both pre-and post-tests. Most of the participants (69.7%, n = 23) were nurses or midwives aged less than 35 years of age. Thirteen (39.4%) had been in service for 5 years or less; and equal proportions of men and women participated in the study (Table 1).

**Table 1:** Participant profile

Characteristic	n (%)
Age (years)	
18–24	3 (9.1)
25–29	9 (27.3)
30–34	9 (27.3)
35–39	8 (24.2)
>40	4 (12.1)
Gender	
Male	16 (48.5)
Female	17 (51.5
Cadre	
Clinical officer	10 (30.3)
Nurse/midwife	23 (69.7)
Years of service experience	
1–5	13 (39.4)
6–10	12 (36.4)
>10	8 (24.2)

### Knowledge scores by cadre

Clinical officers had significantly higher mean pre-and post-test scores than nurses and midwives (p < 0.05). Mean scores for both cadres improved from pre- to post-test; the difference between the mean pre- and post-test scores was 20% for clinical officers and 18% for nurses and midwives. Whereas the clinical officers’ lowest knowledge score improved by 16 points after training, the nurses and midwives maintained the same lowest knowledge score after training, resulting in a non-improvement in the minimum score for the entire sample of participants (Table 2).

**Table 2:** Pre and post training test results by cadre for all participants

	Mean score (95% CI)	Min	Max	IQR	Percentage change^a^	p value (95% CI)
Pre-test						
Clinical officer	64.0 (57.0, 71.0)	52	88	8		0.022
Nurse/midwife	52.9 (47.3, 58.5)	32	84	12		
All	56.2 (51.6, 60.9)	32	88	16		
Post-test						
Clinical officer	76.8 (72.4, 81.2)	68	88	8	20.0	0.002
Nurse/midwife	62.4 (56.9, 68.0)	32	84	16	18.0	
All	66. 8 (62.4, 71.4)	32	88	20	18.9	

^a^Percentage change: (Post-test score−pre-test score/pre-test score * 100)

### Absolute measure of CAMH knowledge

The post-test score was used as the measure of absolute CAMH knowledge after training. Simple linear regression of post-test results at the 5% significance level was performed on all the independent participant factors after ascertaining the normality and homoscedasticity of the post-test variable. Only cadre (p = 0.002) and pre-test score (p < 0.001) significantly influenced the post test result in the crude model. Participant age, sex, health unit, years of service had no influence on post test results (p value >0.05).

During hierarchical linear regression (Table 3), the inclusion of pre-test score into the model (model 2) increased the correlation coefficient for participant cadre, suggesting a confounding effect of pre-test score on cadre and post-test score. Therefore, we re-run the regression using the interaction term pre-test*i.cadre (model 3). The results show a significantly stronger influence of cadre on post-training knowledge than in the crude model: when pre-test score is accounted for, nurses and midwives were 46% less likely than clinical officers to have a high post-test score (p = 0.032). Further analysis with disaggregation of the nurse/midwife cadre shows that this significant result is contributed to by nurses (data not shown): whereas there was no significant difference between midwives’ and clinical officers’ post-test results, nurses were significantly (46.8%) less likely than clinical officers to have a high post-test score (p = 0.018).

**Table 3:** Hierarchical Linear Regression of post-test results

	Model 1 Β-coefficient (80% CI)	Model 2 Β-coefficient (80% CI)	Model 3 Β-coefficient (80% CI)
Cadre	−14.4 (−23.1, −5.7)*	−8.0 (−15.5, −0.4)*	−46.2 (−88.0, −4.4)*
Pre-test		0.6 (0.3, 0.9)*	0.1 (−0.5, 0.7)
Cadre*pre-test			0.6 (0.1, 1.3)

* *p* < 0.05

### Relative measure of CAMH knowledge gain

The change in CAMH knowledge, measured as the percentage difference between pre-test and post-test results was used as a relative measure of CAMH knowledge gain. First, a two-tailed independent samples t-test performed to assess the difference in mean knowledge change between the two cadres showed no significant difference (p = 0.410).

We then divided the sample along three cut-off points of knowledge gain: 10, 15 and 25% gain in knowledge and applied logistic regression to determine the association between NSHW cadre and a gain in CAMH knowledge at these cut-offs. Logistic regression (Table 4) revealed that cadre was not significantly associated with relative knowledge gain at the 95% confidence level for all three selected cut-off points of knowledge gain. Relative knowledge gain was significantly associated with participants’ pre-test score (p < 0.05) at all cut-offs; a higher pre-test score was significantly associated with lower odds of achieving CAMH knowledge gain.

**Table 4:** Logistic regression of CAMH knowledge change

	10% Knowledge gain			15% Knowledge gain			25% Knowledge gain		
	AOR	95% CI	p value	AOR	95% CI	p value	AOR	95% CI	p value
Cadre	0.08	(0.01, 1.19)	0.066	0.16	(0.01, 2.21)	0.170	0.13	(0.01, 1.74)	0.122
Pre-test	0.90	(0.83, 0.99)	0.024*	0.87	(0.77, 0.98)	0.022*	0.87	(0.78, 0.98)	0.018*
Sex	1.72	(0.25, 11.97)	0.582	0.93	(0.13, 6.76)	0.941	0.50	(0.07, 3.48)	0.485
Age	1.21	(0.94, 1.57)	0.143	1.43	(1.02, 1.99)	0.039*	1.31	(0.98, 1.75)	0.069
Years of service	0.57	(0.11, 3.09)	0.514	0.26	(0.04, 1.50)	0.137	0.37	(0.06, 2.17)	0.271

* *p* < 0.05

## Discussion

We aimed to describe an interactive and clinically integrated short in-service CAMH training for NSHW in Uganda and to determine the effect of participant cadre on learning outcomes from such training. Clinical officers had significantly higher pre- and post-training knowledge than nurses and midwives, suggesting that cadre influences pre- and post-training knowledge. However this effect was not observed for three levels of knowledge gain. A higher pre-test score was significantly associated with lower odds of achieving CAMH knowledge gain.

The training in this study met the definition of CME [27]. Further, training was conducted as an interactive and clinically integrated learning activity, which is cited as the most effective form of in-service training for imparting knowledge and skills [27, 37]. Thus, the improved participant knowledge scores after training in this study support the CME theoretical framework by demonstrating that interactive and clinically integrated CME results in improved learning outcomes.

We found that our CME resulted in an overall knowledge gain of 18.8%, consistent with findings reported in other low resource settings. For example, a 35% point change in mental health knowledge was observed for 1000 PHC staff trained in Kenya [38] and in India, a 4 day interactive mental health course resulted in a significant improvement in community health workers’ ability to recognize common conditions [39]. A significant knowledge gain was also observed from mhGAP in-service training of PHC workers in Nigeria [35]. However, those findings were not disaggregated by cadre. A cadre-disaggregated study in Lebanon showed that nurses achieved higher post-training mental health competency scores than doctors following a 12 day training [40], contrary to our finding of lower post-training knowledge scores among nurses than clinical officers. Instead, our findings are similar to those from a mental health training in post-tsunami Sri Lanka which showed better learning outcomes among doctors than among mid-level personnel [41].

The contextual setting of training matters for CME outcomes. The residential CAMH training in our study was conducted at a national teaching hospital, some distance from the city centre, reducing opportunities for late coming and absenteeism while optimising opportunities for clinical practice on the children’s ward. This type of setting is upheld by Bluestone et al. [37] who postulate that a setting that reduces absenteeism and situates the learning in an environment that offers opportunities for clinic-based simulations is critical to knowledge gain. On the other hand, the potentially prohibitive cost of a residential off-site training at a referral facility near the city needs to be considered in planning such a CME in a low resourced-setting like Uganda.

We found that participant cadre was statistically significant for one measure of knowledge (absolute measure) and not for the relative measure of knowledge gain. Specifically, clinical officers were significantly more likely to have higher post-training knowledge scores. This finding seemingly implies that nurses and midwives require more training time and/or different methods, until one considers the fact that clinical officers also had significantly higher pre-training knowledge than nurses and midwives, negating the significantly higher post-training knowledge among clinical officers. The inconsequentiality of the higher post-training knowledge scores among clinical officers is amplified when considered alongside the finding that none of the three set levels of gain in CAMH knowledge were significantly influenced by participant cadre in this study. Thus, our findings when considered together imply that both cadres of NSHW can be expected to achieve equivalent knowledge gain from similar CAMH training.

The evidence on the influence of participant characteristics on CME outcomes is inconclusive for age, gender, years in service or cadre [42, 43] and hinders a discussion on the role participant cadre plays in influencing mental health learning outcomes. Nonetheless, this study agrees with the findings of Lowe et al. [42] who found no significant relationship between participant age, gender and years in service on training outcomes. We attribute the missing link between these characteristics (particularly age and years in service) and training outcomes in this study to a low CAMH patient load at the PHC clinics where the NSHWs routinely practice, which deters the experiential learning that these characteristics are expected to confer. This finding implies that within Uganda’s PHC setting, all NSHW irrespective of gender, age or years in practice would be equally eligible for training as proposed by the existing CAMH policy [11].

We found that higher pre-test scores were significantly associated with a lower change in knowledge. This finding supports Warr et al. [36] who argue that variations in trainee competence before the training will impact learning outcomes and implies the necessity for pre-training screening of potential CAMH trainees, with priority for training given to low-performing NSHWs in low-resourced settings. The relatively higher pre-test scores seen among clinical officers in our study can be attributed to relatively higher exposure to mental health concepts clinical officers receive during pre-service training, especially considering that almost 40% of the participants had been in practice for 5 years or less. It can thus be expected that the knowledge gained in school influenced their level of pre-training CAMH knowledge. However, pre-service exposure to CAMH concepts would not explain the significant difference observed between nurses and midwives with respect to clinical officers’ post-training knowledge. There’s no clear explanation for this discrepancy. Nevertheless, the entire group achieved an 18.8% increase in knowledge from the training, reminiscent of the 18% increase observed in the Lebanese study cited [40].

Overall, the pre-training knowledge was high, as shown by a mean pre-test score of over 50%. This might be due to the nature of the test itself, i.e. one that does not delve deep into psychiatric knowledge, but rather relies on simple true/false and multiple-choice responses to gauge rudimentary knowledge of child and adolescent behaviour. Additionally, the test was a theoretical paper-based one without practical clinic-based assessments. Surprisingly, the mean score increased at the post-test but the range of scores remained the same, indicating that, at least one participant did not improve in knowledge.

Knowledge gain, measured with the relative measure in this study, was uniform across all participant characteristics, confirming that the mhGAP-IG is well suited for non-specialist clinicians in PHC settings, regardless of pre-service qualifications. These findings are consistent with those from a 4 week training based on the mhGAP that showed increases in knowledge across all cadres of nurses and community health workers in Pacific island countries [44]; and agree with Uganda’s policy guidelines which support CAMH training for all NSHW cadres.

We consider the individual nature of the pre and post-test to be a strength of the study, since no discussion between participants was permitted to obscure the results. The small sample size in this study is a limitation. However, the study represents a random selection of level 3 primary care facilities in two districts and we believe these findings can be generalised to all providers in the two districts. Another limitation is that only knowledge gain and not post-training competence of the NSHWs was assessed as a training outcome. A further limitation is that the assessment tool was not set up to identify what parts of the training curriculum were particularly challenging, information which would help in the design of future trainings.

## Conclusion

Non-specialist health workers cadre does not influence knowledge gain from short in-service CAMH training in spite of clinical officers being significantly more likely than nurses and midwives to achieve higher post-training knowledge scores. The study’s main implication for the improvement of human resources for CAMH in Uganda is that nurses and clinical officers are equally capable of improving their CAMH knowledge from short in-service training, in spite of varying exposure to mental health concepts during pre-service training. Thus, an option for the integration of CAMH into PHC in Uganda using the mhGAP-IG and PowerPoint training slides from the IACAPAP textbook is to proceed without cadre differentiation. Based on results from this study, we suggest that selection of training participants in a low resource setting like Uganda should be preceded by a pre-test of all candidate NSHWs, with training priority given to the lowest performing NSHWs; and that training should be interactive and experiential, conducted by highly proficient CAMH practitioners in a teaching hospital setting.

## Additional files

**Additional file 1.** Training curriculum.

**Additional file 2.** Pre and Post-training assessment test.

## Abbreviations

CAMH: child and adolescent mental health
CME: continuing medical education
HC III: health center level 3
IACAPAP: International Association of Child and Adolescent Psychiatry and Allied Professions
IG: implementation guide
LMIC: low and middle income country
mhGAP: Mental Health Gap Action Program
MNS: mental, neurological and substance abuse
NSHW: non specialist health worker
PHC: primary health care
WHO: World Health Organisation
