# Supplementary material for: Does child and adolescent mental health in-service training result in equivalent knowledge gain among cadres of non-specialist health workers in Uganda? A pre-test post-test study
Source: Int J Ment Health Syst. 2017 Aug 24;11:50. doi: 10.1186/s13033-017-0158-y (PMC5571627; doi:10.1186/s13033-017-0158-y)
Supplement: Supplementary file 1 — Additional file 1. Training curriculum. [file 13033_2017_158_MOESM1_ESM.docx]

# Additional file 1. Training curriculum

| **Time** | **Day 1** | **Day 2** | **Day 3** | **Day 4** | **Day 5** |
| --- | --- | --- | --- | --- | --- |
| 8.00 - 9.00am | Registration, introductions, ground rules, expectations, course objectives | **Recap**  The clinical examination of children, adolescents and their families | **Recap**  Managing difficult behaviour | **Recap**  Depression  Suicide + self harming behaviour | **Recap**  Somatoform disorders |
| 9.05- 10.40 am | **Pretest (15 minutes)**  Introduction to child and adolescent mental health and mhgap-ig  Overview of etiology, diagnosis and treatment planning for child and adolescent mental health problems, symptomatology and terminology | General principles of care  Psychosocial treatments  Paediatric psychopharmacology | Autism spectrum disorders,  Enuresis  Encoporesis  Epilepsy | Bipolar disorder  Psychosis  Tic disorders | Physical illness and mental health ( hiv/aids,diabetes, scd, etc) |
| 10.40 – 11.00am | Break | | | | |
| 11.05- 1.00 pm | Normal development- infancy, childhood and adolescence | school underachievementand specific learning difficulties  Intellectual disability | Practical skills  Children’s ward | Anxiety disorders, separation anxiety, ocd, | Alcohol use disorders  Substance use disorders + internet addiction  Evaluation, planning  **Post test** |
| 1.00 - 2.00 pm | Lunch | | | | |
| 2.00- 4.00 pm | Attachment  Positive parenting (parenting capacity and its relationship to attachment and child development)- parental mental illness | Externalising disorders (adhd, conduct disorder, oppositional defiant disorder) | Group discussion of cases | Ptsd  Bereavement | Departure |
| 4.00- 5.00 pm | Group work  Discussion/ tea | Group work using case examples feedback/ planning for clinic day | Group work using case examples feedback | Group work using case examples feedback |  |
