# Supplementary material for: Does child and adolescent mental health in-service training result in equivalent knowledge gain among cadres of non-specialist health workers in Uganda? A pre-test post-test study
Source: Int J Ment Health Syst. 2017 Aug 24;11:50. doi: 10.1186/s13033-017-0158-y (PMC5571627; doi:10.1186/s13033-017-0158-y)
Supplement: Supplementary file 2 — Additional file 2. Pre and Post-training assessment test. [file 13033_2017_158_MOESM2_ESM.docx]

# Additional file 2. Pre and Post-training assessment test

| A. Put **√** in the correct column.  A. Put  in the correct column. | True  True | False |
| --- | --- | --- |
| 1. People with mental disorder usually cannot make decisions concerning their health |  |  |
| 2. People with mental disorder are best cared for in mental hospitals |  |  |
| 3. All people with depression should be treated by antidepressants |  |  |
| 4. Searching for a miracle cure is part of a grief process among children with terminal illness |  |  |
| 5. Providing brief advice to people who have alcohol problems is effective |  |  |
| 6. Mental disorders are common in children and adolescents |  |  |
| 7. To stop acute seizures, diazepam by intramuscular route is the routine treatment of choice |  |  |
| 8. Severe chronic depression in a mother may lead to developmental delay in her children |  |  |
| 9. If the child shows over-activity and inattention, then medication is usually needed |  |  |
| 10. Vitamin injections should be routinely used for somatic complaints with no organic cause |  |  |
| 11. Asking people about suicidal thoughts increases the likelihood of suicide |  |  |

B. Put  **√** for the correct answer. There is only one correct answer for each question.

1. Which one of the following statements concerning depression is correct?
   1. Depression often presents with vague physical pain and fatigue
   2. Depression often presents with delusions and hallucinations
   3. Depression often presents with confusion

1. Concerning antidepressants which of the following is correct
   1. The treatment should be continued even if the person suddenly becomes manic
   2. The treatment should be continued for 2-3 months
   3. The treatment should usually only be offered if the depression affects the person’s daily functioning

1. Which of the following messages should be given to a person with depression?
   1. Try to reduce your physical activity as much as possible
   2. Try to participate in social activities as much as possible
   3. Try to sleep as much as possible
2. A 15-year-old girl says that she hears voices that no one else can hear and is convinced that someone wants to hurt her, which of the following disorders is most likely present

____ a. Psychosis

____ b. Depression

____ c. Mania

1. Concerning the management of acute psychosis

____ a. Medicines by injection will be required for most cases

____ b. The person needs to be followed up at frequent interval

____ c. The person should always be restrained (e.g. chained)

1. Concerning epilepsy, which of the following is correct

____ a. For making diagnosis of epilepsy, first step is to do electroencephalography (EEG)

____ b. Two seizures in last year are reason enough to start antiepileptic medicine

____ c. Once the diagnosis of epilepsy is made in a woman with epilepsy, she should not marry or have children

1. Concerning antiepileptic medications, which of the following is correct

____ a. Antiepileptic medication should be started at a maximum dose and then decreased

____ b. Antiepileptic medications should be combined for faster treatment

____ c. Antiepileptic medication can be stopped two years after the last epileptic seizure

1. After a suicide attempt

____ a. Leave the person alone resting in a quiet room

____ b. Restrain visits from family and friends

____ c. Remove means of self-harm

1. Which of the following statement is correct concerning alcohol use?

____ a. If people drink alcohol every day of the week, they are alcohol dependent

____ b. Alcohol use cannot cause seizures

____ c. People can have an alcohol problem even if they only drink once in one month

1. In Controlled breathing children are asked to:

____ a. Inhale and exhale quickly

____ b. Inhale slowly and exhale quickly.

____ c. Exhale more slowly than you inhale

1. Concerning the management of a child with developmental delay which of the following is correct

____ a. The child should not be allowed to attend a normal school

____ b. Medication can reverse the condition

____ c. Explain to the family that the child can learn new skills

1. Concerning the management of an adolescent with persistent aggressive and disobedient behavior, which of the following is correct

____ a. Provide advice to family and teacher

____ b. Punishment for unwanted behaviors is the best method to improve behaviour

____ c. Medication should be considered as soon as possible

1. Concerning challenging behaviours among children the following is correct

____ a. The behaviour serves an adaptive purpose to the child

____ b. The behaviour can best be managed using psychotropic medication

____ c. Parents need to use corporal punishment sometimes to control behaviour

25. Which of the following statements concerning pharmacological treatment for children and adolescents with mental disorder is correct

____ a. You usually do not need to obtain consent since the person does not understand

____ b. Antidepressants should only be given to adolescents after trying psychosocial treatment

____ c. Once the antipsychotic treatment starts, the person needs to continue taking the drug throughout life
